# Gender at the intersection with race and class in the schooling and wellbeing of immigrant-origin students

**DOI:** 10.1186/s12905-016-0328-0

**Published:** 2016-07-28

**Authors:** Mahsa Bakhshaei, Rita Isabel Henderson

**Affiliations:** 1 Department of Integrated Studies in Education, McGill University, Montreal, Canada; 2 Graduate School of Education & Information Studies, University of California, Los Angeles (UCLA), Los Angeles, USA; 3 Department of Community Health Sciences, Cumming School of Medicine, University of Calgary, Calgary, Canada

**Keywords:** Gender, Race, Class, School success, Wellbeing, Acculturation, South Asian origin girls, Quebec, Structural violence

## Abstract

**Background:**

In French-language secondary schools in Quebec, among all immigrant-origin students, those originating from South Asia have the highest dropout rate. However, girls belonging to this group consistently outperform their male peers of similar ethnic background. This stirs questions about the reasons for this relative outperformance and its linkage with overall wellbeing among these girls.

**Methods:**

A mixed methods approach guided data collection. It involved in-depth interviews with female and male students of South Asian origin (*n* = 19) and with individuals holding educational roles in the lives of youth (*n* = 25). An additional anonymous questionnaire aggregated parent perspectives (*n* = 36), though this article focuses primarily on qualitative lessons.

**Results:**

This article shows three main reasons for why South Asian female adolescents in Quebec French-language secondary schools outperform their male counterparts in schooling attainment: parental expectations after migration, socialization at home, and relationships at school. According to our findings, academic perseverance among these girls does not necessarily translate into their improved wellbeing or their involvement in an advantageous process of acculturation.

**Conclusions:**

This study highlights that although gender, ethnicity, and class can create an interlocking system of oppression in certain social spheres for a specific group of women, it can emerge as advantageous in other contexts for the same group. This provides educational policy makers, as well as school and community workers, with guidance and avenues for action that can promote the wellbeing of immigrant-origin girls through involvement in beneficial processes of acculturation aligned with their improved academic performance.

**Electronic supplementary material:**

The online version of this article (doi:10.1186/s12905-016-0328-0) contains supplementary material, which is available to authorized users.

## Background

The Programme for International Student Assessment’s (PISA) 2012 results indicate a gender gap favouring the school success of girls across Organization for Economic Cooperation and Development (OECD) countries, particularly in high-income countries. As a result, internationally, young women are increasingly more likely to complete their secondary education than young men [1]. This is true across many ethnic groups, such that educational scholars in many immigrant-receiving societies argue that gender significantly shapes the educational outcomes and school experiences of youth from immigrant families.

In the United States, for example, studies indicate that there is increased speed of educational attainment among girls of Asian origin compared to male peers of the same background [2]. Similarly, scholars observe greater participation in cultural activities, as well as commitment to homework and relationships to teachers among Latina girls compared to their male counterparts [3]; lower levels of problem behaviour in schools among Mexican girls [4, 5]; and higher levels in learning outcomes among African and Caribbean girls [6, 7]. Scholars frequently attribute academic gaps between immigrant-origin boys and girls in the United States to poor treatment of boys by school personnel [8]. According to these researchers, evidence of poor treatment emerges in data indicating that immigrant-origin boys in the United States are more likely to perceive school as restrictive, encountering greater conflict with authority figures. Boys also report more racism than their female peers of similar backgrounds, such that gender-based stereotypes influence how school personnel interact with immigrant-origin boys, who are often presumed to be macho and more difficult to teach ([8], p.303).

In France, several studies [9–13] indicate that, compared to girls, boys of North African, sub-Saharan African and Portuguese origin are more inclined towards shorter programs of study, more frequently dropping out of school early to pursue employment, while girls from these backgrounds see schoolwork as a means for achieving independence. Among these same groups, boys are reported to enter the labour market earlier, a transition that is often postponed by girls for as long as possible. Curiously, the expectation and desire to remain longer in both the parental home and in school creates conditions for better academic performance, such that more conservative constraints within the home contribute to higher educational achievement attained by girls from these groups [11].

One problem with such literature is a tendency to presume that more favourable academic outcomes among immigrant-origin girls are a sign of their wellbeing or their involvement in a more advantageous acculturation process. Immigration research suggests that there is a link between patterns of acculturation and immigrant adaptation [14]. According to segmented assimilation theory, the most beneficial form of incorporation into the host society, especially for second-generation youth in disadvantaged contexts, is “selective acculturation” [14, 15]. This occurs when both parents and children adapt to the mainstream society and pursue upward social mobility while preserving cultural ties to the community of origin. An unintended consequence of more favourable academic outcomes among immigrant-origin girls is that their seeming academic outperformance may mask family, community, and school dynamics that undermine their healthy acculturation and restrict their wellbeing. This literature leads us to question explanations for academic gender gaps among immigrant-origin students that measure the degree to which each gender conforms to educational systems in a host society. Arguably, attention is needed for how family, community, and school dynamics are experienced according to one’s gender and racial background, as these dynamics influence acculturation processes and in turn wellbeing.

Students of South Asian origin (mainly from India, Pakistan, Bangladesh, and Sri Lanka) in Quebec’s French-language secondary schools are an immigrant sub-group of particular interest in this regard. This is because, when compared to all other immigrant regional sub-groups in the province, they display not only the largest gap in female versus male school performance but also the highest dropout rate from school [16].^1^ Today, nearly 25 % of Quebec’s schoolchildren under the age of 18 are either immigrant themselves (first generation) or the children of at least one parent who is an immigrant (second generation).^2^ Quebec’s education system has both French and English sectors. As outlined in the province’s 1977 *Charter of the French Language*, in the public education system the majority of immigrant-origin students are required to attend the French sector until the end of their secondary studies. As a result of this law, nearly 90 % of immigrant-origin students in Quebec are currently enrolled in the French sector. In some of Montreal’s public French-language schools the number of immigrant-origin students reaches as high as 70 % [16]. In such schools in 2012, while 37 % of students were French speaking as a first language, and 21 % English speaking, 42 % had neither of these languages as a mother tongue or language used at home [17].^3^

This article aims to answer why first and second generation female adolescents of South Asian origin in Quebec’s French-language secondary schools significantly outperform their male peers of similar ethnic backgrounds. In answering this, it also seeks to explain whether, as the literature would suggest, such outperformance may translate into involvement in a more beneficial acculturation process and greater wellbeing for these girls. To this end, we look at the socio-educational experiences of these students—namely social and educational experiences lived in their families, ethno-cultural communities, and schools—drawing on the perspectives of South Asian students, their parents, teachers and community workers. Socio-educational experiences are often sewn together to construct a scheme of adolescents’ world and the individual's place in it ([18], p.133). This study uses the term “educational experience” rather than “schooling experience,” as education includes a range of experiences beyond learning enacted through a school curriculum [19]. Therefore, in addition to curricula and pedagogy, “educational experience” refers to an individual’s relationships with teachers and peers in school. In this article, wellbeing refers to quality of life and includes both emotional reactions and cognitive judgments [20]. Following Portes and Rumbaut [21], acculturation refers to a multidimensional process consisting of the confluence between one’s culture of origin and receiving culture. Throughout the paper, we are concerned with signs that indicate the involvement of immigrant children in a selective pattern of acculturation, as defined by Portes and Rumbuat [21], particularly: minimal parent-child conflict, preservation of parental authority, valuable skills of biculturalism and bilingualism, and higher self-esteem among youth.

This study also addresses two additional gaps identified in literature on immigrant youth. First, while recent scholarship across many ethnic groups in multiple immigrant-receiving societies has consistently shown strong gender differences favouring girls in educational outcomes, why and how gender makes such a difference has remained relatively unexplored in diverse contexts, including in the United States [8, 22] and in Canada [23, 24]. Second, this study responds to Qin’s [22] call for studies that explore the intersection of gender, ethnicity, and social class, and how this intersection impacts immigrant children's education and adjustment. We base analysis in the race-gender experiences theory, as posed by López [3], to examine what the intersecting dynamics of race and gender reveal about educational outcomes, acculturation patterns, and wellbeing among South Asian origin adolescents in Quebec. According to this theory, intersecting experiences of race and gender have a cumulative effect on the schooling and prospects for social mobility of immigrant-origin students. Race-gender experiences involve “episodes in which men and women undergo racial(izing) and gender(ing) processes in a variety of social spaces, including but not limited to public spaces, schools, work and family life” ([3], p. 69). These experiences extend beyond school performance disparities, as differential access to education and employment may shape health and resiliency well into adulthood. As we discuss in more detail below, South Asian communities in Quebec encounter serious socio-economic challenges. Educational research in the province shows that among the whole population, social class more than gender seems to play a role in school dropout [25, 26]. As such, in this study we cannot ignore the socio-economic status of South Asian families in Quebec; throughout, socio-economic status is an omnipresent element that defines many particularities in race-gender experiences of South Asian youth in Quebec. Arguably, these experiences can be different for girls within similar ethno-cultural backgrounds who come from distinct socio-economic contexts [27].

Race-gender experiences as a theoretical framework poses unique insight for South Asian women and girls in Canada. Certainly the socio-cultural experiences of South Asian adolescent females in migrant contexts have involved many South Asian families adopting Western cultural values and practices. Nonetheless, researchers have observed that the maintenance of traditional gender roles among South Asian families, particularly among those with low socio-economic status either in the home country or abroad, is not uncommon in North America. This is true to the extent that adolescent girls interviewed for one study in Montreal “felt that their parents and communities have more stringent rules for female socialization than any other community in Canada” ([28], p.615). For these scholars, collectivist and patriarchal values are observed in male relatives controlling the social activities of daughters, sisters, and female cousins, as well as in the persistence of arranged marriages following migration. Scholars have also identified that in some South Asian families, any perceived violation to such values may be met with swift repercussions within the home, including but not limited to threats and actual physical violence against girls [29, 30]. It should be noted that, at the time of data collection for this study, the Shafia murder trial was prominent in the Canadian media, addressing the honour killing of four female family members from a South Asian country by relatives [31]. One report out of the University of British Columbia identifies domestic violence as a leading cause of suicide among South Asian women, whereby these are more likely to report sexual health concerns and have greater risk for depression and anxiety than other female groups [32]. Such studies indicate that South Asian women who are raised in Canada face particular challenges in negotiating diverging expectations of them; they must reconcile societal expectations that emphasize creating an independent identity that is competitive in the workplace, with domestic expectations that call for the preservation of one’s cultural heritage by affirming prescribed gender roles and collectivist family values [28, 30, 33]. Tensions resulting from these competing identity expectations have been noted to cause stress and confusion, as many women struggle to belong in both worlds, yet wind up not fitting entirely into either [34, 35].

The concept of “structural violence” is also used here as an analytical framework for observing how race-gender inequities are not restricted to traditional practices specific to certain ethnic groups, but rather embedded in wider social, political and economic structures. Sociologist Johan Galtung [36] coined the term to refer to a form of violence wherein social structures or institutions may harm people by preventing them from meeting their basic needs. In other words, Galtung used the term “structural violence” as a call for social justice, to identify the means by which certain groups who monopolize resources benefit from prevailing systems, impeding others who are denied resources from overcoming isolation and oppression. We argue that structural violence endured by South Asian girls in Quebec is a social injustice that requires extending attention beyond conventional dichotomies (e.g., victims vs. perpetrators of patriarchy), such as about who is responsible for producing the race-gender gap and how.

The multi-disciplinary backgrounds of the co-authors bridges two fields interested in the daily experiences of marginalized youth: the sociology of education and population health promotion. Each of these fields pays close attention to the short-term and long-term consequences of structural inequities. This is because evidence indicates that, at great cost to society, preventable inequality not only increases unemployment, poverty, poor housing, and ill health, but also social suffering [37], this last referring to the cumulative lived experience of harm. Importantly, advocating for a preventive approach for health grounds the concept of wellbeing in a social determinants framework, whereby health is not the absence of disease but rather, to quote the constitution of the World Health Organization, a “state of complete physical, mental and social wellbeing” [38]. For immigrant-origin girls, health promotion is therefore as much about access to adequate healthcare as it is about ensuring supports within the social spheres they occupy for meaningful integration and participation in society. This approach highlights that while relative academic achievement may be a sign of healthy acculturation and wellbeing for some immigrant-origin youth, it may also signal marginalisation.

### South Asians in Canada and Quebec

The term South Asian describes people of various religions and nationalities who trace their cultural origins to the Indian subcontinent. South Asia includes Afghanistan, Bangladesh, Bhutan, India, the Maldives, Nepal, Pakistan and Sri Lanka [39]. Given the prevalence of migrants from certain countries of origin, as mentioned above, our study is limited to students originating from India, Pakistan, Bangladesh, and Sri Lanka. As of 2011, over a million and a half individuals in Canada self-identified as South Asian [40]. Of diverse cultural, religious, and linguistic backgrounds, these individuals compose the largest regional visible minority group^3^ in Canada. Great diversity exists within this population with regards to religious affiliation, language, immigration history, socioeconomic status and education. Despite their differences, South Asians generally share some common characteristics, including values around family expectations and beliefs that relate to education and wellbeing. Common values have been noted in the literature to include collectivism and primary allegiance to the family, self-sacrifice, patriarchy, and respect for elders [39, 41].

The South Asian community in Quebec is relatively new to the province. In 2011, nearly 90,000 South Asian Canadians lived in the province, mostly in the major city of Montreal. The Ministry of Immigration, Diversity and Inclusion (MIDI) indicates that nearly 48 % of this population is female [42]. Sociological studies show that this population faces considerable challenges to economic, linguistic, and social integration [43, 44]. For instance, the majority of South Asian immigrants speak a mother tongue other than French or English, and in general they master English more than the province’s dominant language of French. The employment rate for South Asian immigrants in Quebec is lower compared to the overall Quebec population, and unemployment rates are nearly double [42]. South Asians also live more segregated and insulated from the dominant society than other immigrant groups [43]. Spatial concentration arguably frames greater difficulty experienced in terms of economic and linguistic integration [44]. Meanwhile, several studies confirm that members of the South Asian community are frequently discriminated against in Quebec for reasons related to outward physical appearances, including for perceived religion and skin colour [45].

Despite these challenges, South Asian immigrants in Quebec have not been studied by social scientists to any appreciable degree compared to other visible minority groups grappling with immigration and integration. The literature on South Asians (limited to the four countries of origin mentioned already) has mostly examined identity and cultural transmission within families [28, 30, 34], transcultural clinical issues among refugees [46, 47], social and psychological problems related to women [48, 49], and health concerns [50–52]. Contributing a socio-educational analysis to this literature, this study renders a more holistic, cross-disciplinary picture of wellbeing.

## Methods

Data presented here were collected as part of a larger mixed methods (qualitative and quantitative) research project exploring family, community, and systemic dynamics that influence the socio-educational experiences of South Asian origin students in French-language secondary schools in Montreal [53]. Data were collected throughout 2011 and 2012 by means of semi-structured qualitative interviews and a quantitative survey in three domains: in schools, in the community, and among families. The Université de Montréal’s Multi-Faculty Ethics Review Board approved qualitative and quantitative instruments related to this investigation, which included consent to publish data provided by all participants, as well as by parents for under-aged participants.

### Qualitative interviews

A total of thirty-five individual or paired interviews were carried out in all of the three domains, involving forty-four participants (*n* = 44). School-based data collection occurred in partnership with two Montreal secondary schools with a significant concentration of South Asian students. Both had low socio-economic status, being located in two low-income multi-ethnic neighbourhoods inhabited by a large number of South Asians. Both offered “welcome classes” for students who were not French speakers. In the schools, we recruited 17 teachers and non-teaching personnel (*n* = 17, see Table 1), as well as 9 female and 10 male students of South Asian origin (*n* = 19). Teachers taught different courses (e.g., English, math, and history) and included welcome class instructors who have unique exposure to immigrant-origin students and families during their first months in the province. Non-teaching personnel included principals, assistant principals, social workers, psychologists, career-related program coordinators, coordinators of intercultural relations, and counsellors in charge of dealing with student delinquency.

**Table 1:** Distribution of professional backgrounds among school personnel (*n* = 17)

Professional Role	Number of Participants
School administration—principal/assistant principal	3
Teacher	8
Social, health & mental health service provider—social worker; psychologist	3
Youth development worker—career-program coordinator; intercultural relations coordinator	2
Delinquency counsellor	1

Students included male and female (See Table 2), first and second-generation youth from the four countries indicated between 15 to 18 years of age. They all came from families with low or middle socio-economic status living in the surrounding neighbourhoods in which the schools were located. It is worth noting that upon learning that the first author originates from Iran, the interviewed girls often seemed to feel more comfortable sharing details about their personal lives. This may be a result of considering her as someone who shared similar experiences as them, and for whom keeping up one’s community reputation was not needed.

**Table 2:** Profile of student participants (*n* = 19)

Country of Origin	Gender		Generation	
	M	F	First	Second
India	4	4	2	6
Pakistan	1	1	2	0
Bangladesh	3	2	3	2
Sri Lanka	2	2	3	1

Community-based data collection occurred in partnership with four organizations that were either explicitly South Asian community associations, or centres committed somehow to the social or educational development of immigrant-origin students, including youth from South Asia. All four organizations were located in the same neighbourhoods as the partnered schools, and as such catered to a large number of South Asian students from the schools. Two were specifically South Asian community associations. One was an after-school tutoring centre where we interviewed a sciences and math tutor originating from an immigrant family with several years of experience working with South Asian children. The other was a South Asian Women’s Centre where we interviewed a stakeholder working with girls on empowerment projects. The two other organizations had the mandate to contribute to the personal and social development of youth by way of educational support services, individual and group guidance, and the organization of cultural and recreational activities. These organizations were also tasked with sensitizing parents to youth needs and the realities in which they live. The director in one and a service provider in the other were interviewed, the latter directly collaborating in an after-school tutoring program with welcome class instructors at one of the partnered schools.

Meanwhile, data collection involving family members included mothers and fathers from different socio-economic statuses in Quebec who originated from India, Pakistan, and Bangladesh (See Table 3). Recruitment of parents into the study was fostered by the support of the targeted community organizations. Recruitment criteria for these was that they had lived in Montreal for at least five years, currently had a child attending a French-language high school in Montreal, and spoke French or English. Their children’s place of birth was not a criterion taken into consideration. In spite of efforts over a period of seven months to recruit more to interview, it was only possible to complete interviews with four parents, and only one among these had a student who attended one of the partnered schools.

**Table 3:** Profile of interviewed parents (*n* = 4)

Mother/Father	Home country	Level of education	Current occupation	Occupation in the hom country	Child	
					Sex	Place of birth
Father	Bangladesh	Master’s	Engineer	Engineer	M	Canada
Mother	Pakistan	Master’s	Community worker	High school teacher	F	Canada
Mother	Pakistan	College	Housewife	Housewife	F	Canada
Mother	India	High school	Labourer	Housewife	M	Canada

### Quantitative survey

Given the limited number of parents recruited for interviews, an anonymized closed-ended questionnaire was created to better solicit perspectives among this stakeholder group. Recruitment criteria were also broadened to elicit more responses. Criteria included: being of South Asian origin, having moved to Montreal sometime in the past fifteen years, currently having a child attending a Montreal French-language high school or having a child that had completed or dropped out of one within the past fifteen years. The survey involved fifty Likert scale yes/no and semantic differential scale questions (See Additional file 1. Questionnaire (for parents). The Educational Success of Quebec Youth Originating from South Asia). It proved better adapted to the availability of parents who, for linguistic barriers, time constraints, or reluctance to participate in recorded interviews, were more responsive to sharing their views by way of filling out an anonymous questionnaire. Thirty-six (*n* = 36) relevantly completed questionnaires were returned.

Both quantitative and qualitative instruments focused on the socio-cultural profile of parents; the pre-migration and migration circumstances of families; hopes among parents for their children’s education; perceived contrasts between South Asian values and those promoted in Quebec; the organizational structure of South Asian communities; relationships within families, schools, and the broader community; and social, academic, and systemic dynamics shaping school experiences. The diversity of perspectives shared in the questionnaire highlights a challenge for the authors here, to represent the complexity of experiences among South Asian parents and students who expressed both similarities and notable differences in their cultural backgrounds, socio-economic status, level of acculturation, and individual personalities.

### Data analysis

A structured analysis of qualitative data involved two steps. It began with segmentation and decontextualization of data, followed by categorization and recontextualization [54]. After having transcribed the audio recordings of interviews, the texts were read and re-read in order to best grasp their content. Once themes were identified, recontextualization began, involving taking analytical categories, restructuring these, and identifying meaning in relation to the research questions and with the study’s theoretical framework. Given the limited number of questionnaires gathered in the quantitative portion, it was not possible to carry out a regression analysis, which might have enabled correlations between multiple variables. Nevertheless, data were analysed descriptively, though they remain limited to the response categories provided in the original questionnaire. In the end, the qualitative approach offered greater sensitivity to a wide range of themes that were not as easily addressed in the questionnaire, including complex topics like parental control and the heterogeneity of responses. For this reason, the majority of data relevant to the question of race-gender experiences discussed here are qualitative. Quantitative data emerge primarily to complement qualitative sources, either supporting analysis or enhancing the context [55].

## Results

Supporting quantitative research in the area [17, 56], the majority of interviewees who work in schools described a marked difference in the academic performance of boys and girls from South Asian countries. At least six teachers described these girls as “*harder working*” and “*more disciplined*” than their male peers of similar background. One English teacher described South Asian female students as “*little girls* (…) *who are extremely polite, perform well enough, and who work very, very hard*”. A history teacher observed that these girls are “*always very discrete, and always ready*”. Nevertheless, teachers contrasted their positive impressions of South Asian female students with observations of “*certain unique experiences*” (History teacher) among these girls. The nature of these experiences may be gleaned from the interviewees’ statements, including from the girls themselves.

### Closely monitored and compliant

Almost all of the parents that participated in the quantitative survey either “agreed” or “strongly agreed” that they have no problem with Quebec schools encouraging their daughters to adopt more liberal behaviour than what is expected of girls within their communities of origin. However, qualitative interviews with different participant groups (including with parents) suggest that the greatest cultural anxiety within families of South Asian background is with regards to their daughters’ exposure to socio-cultural influences from outside their communities. Consistent with results from other studies in Quebec [28, 33] and elsewhere in the South Asian diaspora [15, 57, 58], our interviews indicated that among South Asian communities in Quebec, collectivist and patriarchal values tend to treat women and girls as representative of the level of attachment that such families have to the home country’s culture. In this context, female kin are perceived to carry an important part of their families’ honour. This was observed in encounters between school workers and South Asian families. One social worker explained for this study that she understood that a girl seen by relatives to have behaved “*inappropriately*” may as a result be considered as having “*tarnished the family’s name*”.

Being the face of family honour was often seen to play out as teenage girls encountered greater controls and less freedom than male kin regarding clothing, social relationships, and everyday activities (e.g., going out, speaking with members of the opposite sex, socializing on Facebook, and attending public events). Several of the youth interviewed, including boys, spoke about this reality. Notably, this control over the autonomy of girls appeared more prominently in families having recently arrived in Canada than among those with children born in Canada.

*We don’t face the same consequences, that’s for sure, because we are “guys”* [gestures quotation marks]*, nobody worries. For example, we can take off as many clothes as we want. We can wear something as short as we want. We can go out at night, and almost nothing will happen. Meanwhile, if you are a girl and you take off a little bit of clothing, you will pay for it. This means that guys are left to do what they want because everyone knows they can figure their way out of things, and plus they are “just boys”* [gestures quotation marks]*. So nobody really gets bothered with the guys, but with the girls, no way! They are controlled. It’s right after school, at home! No hanging out!* (Male adolescent originating from Sri Lanka)

Control over the public lives of the girls was exercised mainly by men in the family, and this not only by fathers and older brothers, but also sometimes by younger brothers and even cousins. Moreover, this was not limited to the social relationships of girls, but also extended to their schooling and academic activities. Some participants described girls being escorted daily between school and home, or being monitored at school by a brother or a cousin who took note of the social relationships and grades of female relatives. Some school professionals speculated that this seemed more common specifically among families of Islamic background.

*She was having an issue with something. We had left the classroom and I was arguing with her outside when her brother walked by. He was two years older than her and asked me if there was a problem with his sister. He asked me to tell him what had happened because he was the family authority. He said that he was like a second father and that I was obligated to tell him what had happened. He told me that regardless of what may have happened, at home both he and his father would confront her about it. He also said that he was responsible for her because he drove her to and from school every morning and evening.* (Counsellor in charge of preventing delinquency)

According to some interviewees who worked in schools, relatively strict upbringing was believed to cause what they described as “*passivity*” among many South Asian girls, especially recent immigrants. One teacher felt that this prevented the girls from “*questioning, projecting for the future, and taking advantage of freedoms they have here*” (Teacher of welcome classes for under-educated students).^4^^,^^5^ This teacher went on to describe what they called a “*culture of silence*” promoted among South Asian families that was seen by outsiders to frame the attitudes of girls towards school and social life, where “*they never speak up, even when they have the right answer.”* Another teacher of welcome classes for under-educated students observed that some newly arrived South Asian girls were not accustomed to making choices. This was seen to reflect competing expectations faced by the girls between school and home life:

*It’s a shock for them to arrive here and be expected to perform in entirely different ways* […] *Often girls, especially Pakistani girls, at the start of the year, I ask them on purpose to pick between a blue or red pen, I ask them: ‘Choose, come choose, choose which one.’ And they are like ‘Oh, no, no!’ At the start, even choosing the colour of a pen is really difficult* […] *Make up a character, an alien. You give him a name, what’s his name?’* [And her answer] *‘Hmmm…’ That’s difficult*. (Welcome class teacher)

Some school personnel compared the behaviour and attitudes of recently arrived South Asian girls to those of the generation of their own grandmothers from Quebec. This comparison enabled these staff members to empathize with the perspectives of the young girls, while anticipating teaching methods and modes of socialization appropriate for promoting adaptation to Quebec society.

As demonstrated in other studies on South Asian families in Canada and the United States [15, 25, 30], another strategy among South Asian immigrant parents in Quebec to keep their daughters attached to their culture of origin was to arrange endogamous marriages, preferably with men from their own culture and especially their own religion.

*He has to be Indian and Sikh, either in India or born here, in England, or anywhere. But he has to be Sikh, with a family name to show for it.* (Female adolescent originating from India)

Facing new realities and opportunities in the new country, female teenagers often felt that restrictions in their social life were, in their words, “*unjust.*” While these restrictions were often considered normal by their parents, and described as cultural traditions, the girls explained that restrictions were routinely the subject of debate and conflict within the household. Many of the girls felt they were torn between two worlds with polarized values; in school they were taught values emphasizing individual autonomy, voice and self-determination, whereas at home they were taught the importance of conformity and sacrifice.

### Self-harm and secrecy

As a result of being pinched between distinct expectations, South Asian girls were seen by many school professionals to grapple with a loss of identity and social landmarks. This was believed to shape feelings of isolation, stigma, stress and depression among the girls. According to one social worker, *“they close up to endure alone what they have to live.”* Several school staff members reported having observed self-harming behaviour among South Asian girls. This included an example of one girl having slept outside for more than half an hour in the snow in -30 °C, while other girls went without food for several consecutive days. One school counsellor speculated that such behaviour was rooted in pain believed to be endured by the girls, “*connected to their culture shock or their experience of being controlled and pressured*” (Counsellor in charge of preventing delinquency). It is worth noting here that this corresponds with observations from other Canadian researchers, who have reported a high rate of psychosomatic illness (i.e., bulimia and anorexia) and suicide among young females of South Asian origin [32, 57, 58].

*For South Asian* [girls]*, you see a lot of self-harm, like they will stop eating. For example, I work with one who decided to stop eating. It has been two and a half weeks and she hasn’t eaten anything, and she won’t work either. Things aren’t going well at home and she is seeking attention. She is Pakistani and her boyfriend is Indian; they are from two different religions even. Her parents don’t know she has a boyfriend and they want to marry her to someone abroad; they have already bought the plane tickets.* (Counsellor in charge of preventing delinquency)

Several other girls perceived a high familial and social cost to resistance. They told the research team that they aligned their behaviour with their families’ wishes in order to avoid conflict at home. For example, many girls described avoiding walking with boys at school, because if any of their relatives or family friends saw them, word could get back to their parents. As a result, the girls could potentially be held responsible for undermining the family image, which could in turn create further problems for them. By and large, the girls accepted these conditions and hoped to change the situation in a gradual fashion.

Some girls were known by school staff to carry out parts of their lives in secret from their families. For instance, school workers described that many Muslim girls of South Asian origin remove their headscarves upon arriving at school, while some were even known to be in secretive romantic relationships. Sometimes, out of fear of retribution, the romantic relationships were hidden from everyone, even peers at school, limited to simply text messages. Referring to their parents, some girls identified as culturally distinct from older kin:

Interviewer: *Why do you think you have to hide it?*

Bangladeshi-origin girl 1: *I don’t think they will understand my decision.*

Bangladeshi-origin girl 2: *Their culture is different from ours. They grew up in another country, and in the place where they grew up, there aren’t the same conditions as in our country. They grew up in a conservative country, and here everyone is open; boys, girls, we talk, we hang out. But in their country, it’s the boys on one side and the girls on the other.*

Interestingly, these two girls (one who was born in Canada and the other who had arrived in Canada at an early age) associated their culture with Canada and dissociated themselves from their parents’ culture and country. As this last excerpt illustrates, social restrictions and lack of freedom can alienate girls from their parents. This alienation was observed throughout interviews with the girls as they described differences between life inside and outside of the home, particularly in terms of contrasting cultural practices and values. The girls spoke about how this created an opposition between “us” (youth raised in Canada) and “them” (parents from South Asia).

Several professionals working with this population believed that the hesitation among South Asian girls to seek outside help stemmed from fear of tarnishing the family honour. This complicated the ability of social workers to make on-site visits to family homes.

[The girls ask for] *a degree of confidentiality.* [They ask] ‘*will this remain between us?’ It becomes quite complicated, such as with one girl I remember, who lived in a building full of people from Bangladesh, where they would all know if a social worker had come to visit. They would watch from their windows and we could risk tarnishing* [the girl’s] *honour a bit. I remember with one case, this was a real issue and it had significant repercussions for the girl. It was really difficult.* (Social worker)

This social worker expressed concern that withdrawal from help-seeking behaviour is likely to further isolate such girls from supports to ensure their wellbeing.

### Stereotypes and stigma

The interviewees who worked in community organizations, as well as parents who completed the questionnaire, predominantly indicated that they felt the opportunities offered in school to South Asian girls were not always reflective of the students’ abilities. Community workers argued that reduced expectations from educators amounted to prejudice, as it prompted teachers and staff to envisage limited possibilities and potential for South Asian girls.

*A lot of counsellors, when they speak to “South Asian girls”* [gestures quotation marks]*, are not very open necessarily. And so there is this closed mindset that ‘Well you know what, we could give you these options but you’re probably not going to use them.’ And it’s something that I’ve seen in high schools* (…) *I don’t want to use the word prejudice because I feel like I’d be generalizing, but it’s what it feels like sometimes. Because there is this idea in the back of your mind about what it means to be a “South Asian girl”* [gestures quotation marks] *if you’re not a South Asian*. (Community worker)

The community worker’s usage of quotation marks alludes to a tendency among school staff to perceive South Asian girls through an essentialist lens. The prevalence of stereotypes caused many school staff to assume the girls would not be pursuing higher studies, and prompted staff to reduce the resources provided to such girls. Some participant teachers, community workers, and parents considered that lowered expectations of South Asian girls reflected limited knowledge among teachers and school staff regarding socio-cultural characteristics and the pre-migration situations of South Asian families and children.

Moreover, several teachers and non-teaching personnel believed that this lack of knowledge about certain backgrounds likely resulted in the adoption of two common approaches among educators when they receive newly arrived students. The first approach involves an over-identification of students from certain ethnic groups (including South Asians) as “under-educated” in their learning, resulting in the over-representation of these groups in classes for students deemed under-educated.^5^ The second involves the under-diagnosis of students from certain ethnic groups (again, including South Asians) as having learning difficulties. This trend emerged in interviews as the professionals admitted to being apprehensive about accusations of racism should they assess certain immigrant-origin students as “learning impaired.”

*In the school board, when a student goes for an evaluation exam to see if they have been sufficiently schooled or not, there is an issue that arises in the lack of systematization. For some countries, they don’t test the students. We take for granted, for instance, that a child from Eastern Europe has been schooled, that a child from China and other countries has been…Perhaps they say ‘Oh, here, Mexico. Ah, he might not be schooled enough.’ So they go and do an evaluation with that* [country of origin in mind]*. So, sometimes you have kids that get* [classified] *as below their grade level that in the end haven’t been evaluated* [for learning difficulties]. (Welcome class teacher)

As a result, students with actual learning difficulties may either lose their motivation by being placed in classes that are under their level of knowledge and skill, or simply do not receive the added support that they need.

### Relative academic success

In spite of close monitoring on the part of male kin, cultural stress leading to self-harming behaviour, and lowered expectations on the part of school personnel, girls of South Asian origin in Quebec’s French-language secondary schools tend to outperform and be more disciplined than their male peers of similar background. For instance, some teachers perceived that the tendency among family members to control and limit young female kin made these girls behave more responsibly than boys. This was in turn believed to have played a positive role in their relative academic success. After all, being responsible and serious are features considered in the educational scholarship to facilitate success in school [56]

[These] *girls occupy the role of mom at home, often taking over. They are very very mature for their age.* (History teacher)

Another explanation put forth by school personnel was that, given that girls are under more parental control than their brothers, resulting in spending more time at home, the former could have more positive feelings towards school. This view proposed that the school was among the few spaces of freedom and socializing:

*School becomes the site where their life happens, and other students in the class become their best friends. They don’t have permission to go out. Their only place for socializing is at school.* (Teacher of a welcome class for under-educated students)

The notion that girls and women represent the family honour was also perceived to play a role in the greater success of girls of South Asian origin. The stakeholder interviewed at the South Asian Women’s Centre attributed the greater academic success of girls from her community to the higher expectations parents had of their daughters over sons.

*There isn’t as much pressure on the boys, whereas for the girls it’s like, the girl is the honour of the family. So we’ll project everything onto the girl, whether it’s—you know—what she’s wearing, what she’s doing, who she’s talking to or what her grades are…It’s definitely part of patriarchal society and community, which exists all over the world but in South Asian communities is more prominent.*

Expectations around a son’s place in the family and his future role as a “*second father*” supported this perspective and revealed norms of socialization which may lead some boys to be more invested in family leadership than in academic success *per se*. As a result, the academic success of boys of South Asian origin could also be aligned with an explanation offered in educational psychology literature internationally, whereby the academic failure of boys treats school as mostly a “preoccupation of girls” [59], something which boys from low socio-economic status also often affirm. Arguably, certain aspects of patriarchy may further devalue schooling for boys. This emerged in the relationships that these boys sometimes appeared to have with their female teachers, in spite of evidence that authority is strongly valued in South Asian cultures [39, 41]:

*Sometimes there are issues with authority. I’m a young woman and therefore with boys, when I have to discipline them—*[like] *give suspensions or when I have to call their parents—the boys clam up, having nothing to do with me*. (Counsellor in charge of preventing delinquency)

A final explanation for the relative academic success of South Asian girls lies in the greater consequences that could befall them in comparison to boys should they not perform well. Examples of consequences cited by girls interviewed in this study included being forced to withdraw from school, being forced into a marriage, or being involuntarily returned to a country of origin. A few girls also indicated that in some cases the reverse can also be true, that whether vocalized or not, success in school may give their family a better chance to secure for them a more favourable marriage.

*When boys drop out, it is to help their families. And when girls drop out, the consequences are graver. We see it a lot at school. For instance, when we meet with parents to let them know that something is not going well with their child. If it relates to their daughter, their reaction is always: ‘We’ll deal with this at home!’… It’s a lot of stress. The girls try to do their best in order to show their parents that they are capable of succeeding and doing well in life even if they don’t marry the ideal person.* (Counsellor in charge of preventing delinquency)

Explanations for why South Asian girls outperform their male counterparts in Quebec French-language secondary schools are influenced by what many educators and South Asian immigrants alike consider to be cultural norms and practices related to the South Asian community, particularly around collectivist patriarchal values. However, evidence that South Asian girls display the ability to outperform male counterparts in spite of prejudice, lower expectations put on them, and limited resources extended to them by school practitioners indicates resiliency among these girls to defend their interests in the face of competing pressures between home and school. This resiliency invites deeper reflection on the significance of their academic achievement both within their life trajectories and in terms of services extended to visible minority girls in migrant contexts more generally.

## Discussion

This study finds that a gap in academic results between male and female adolescents originating from South Asia in French-language secondary schools in Quebec can be in part explained by particular experiences that girls encounter at both home and school, formed at the intersection of gender with ethnicity and socioeconomic status. The shared voices of students, parents, and educational professionals indicate that becoming accustomed to a western individualistic culture can be a challenge for some South Asian families in Quebec, who at the same time encounter major obstacles in their social and economic integration. As a result, they may continue to emphasize selective gender-based values from their country of origin. Consequently, girls’ behaviour may be controlled by intense community scrutiny both inside and outside school. A similar dynamic has been identified among Yemeni American girls in a suburb of Detroit [60]. Here, school is not only a domain in which these youth evidently aim to perform well, but one that creates for girls of South Asian origin in Quebec one of the few acceptable settings for social life beyond the watchful gaze of parents, families, or even members of their ethno-cultural communities, where the girls often have few friends or connections to the host society.

Additionally, within migrant contexts, scholars have noted that young South Asians are under pressure to act according to the values of the community of origin [61, 62]. Among families where women are often seen to be responsible for housework, passing on cultural traditions, as well as caring for children, the elderly, other relatives and close friends, rules for young women can be particularly stringent, where the honour of the family can be based on their behaviour and social activities. Thus, scholars observe that girls and women in these contexts tend more frequently to submit to the position of men, who are more often held in the home to be breadwinners, decision-makers, and authority figures [61–63]. Cultural values encouraging female submission and the nurturing of others also turn out to, at times, play a positive role for girls, for example through a sense of responsibility for doing well in school. Concerned about the prospect of an arranged marriage or of being returned to one’s country of origin, some girls are further motivated to display perseverance and academic success, anticipating that they may ensure for themselves a better future with a successful marriage should they excel at school.

While research indicates that education is generally valued among South Asians in Quebec [53, 59, 62], data here suggest that among families of South Asian students in French-language secondary schools in Quebec, parental expectations of academic performance are generally higher for girls than for boys, elevating the likelihood of a performance gap. Data from this study suggest that this can be partly explained by the notion that girls and women represent the family honour. However, we should not forget that the South Asian community in Quebec generally encounters serious socioeconomic challenges, and that the two partnered schools are located in neighbourhoods with a high concentration of poverty. Their relatively low socio-economic status compared to the overall Quebec population, may play a role in the maintenance of traditional gender roles among these families, potentially reinforcing the role of boys as breadwinners and elevating the likelihood of a gendered performance gap. This corresponds to other studies in Quebec that indicate that social class more than gender leads to school attrition, where boys more than girls from under-privileged areas adhere to conservative gender stereotypes [25, 26].

The findings demonstrate that gender inequities are not perpetuated by immigrant ethno-cultural groups alone, but also by prevailing structures within the host society, namely socio-economic factors and racial dynamics. This is evident when South Asian girls display resiliency in their school performance in spite of unfair treatment upon arrival in Quebec’s school system. It is observed through inappropriate assessment of prior learning motivated by race-based prejudices, as well as during their schooling in Quebec’s system, where these girls frequently encounter lowered expectations extended to them that are motivated by such race-gender prejudices. Research shows that student inclusion and graduation rates are strongly correlated with teacher expectations of their students [63, 64]. When students are not engaged or feel that teachers do not respect their rights and individual or collective identities, youth may seek validation in oppositional behaviour, such as attrition, drug use, gang membership or even involvement in terrorist activities [65].

What all this means for the wellbeing of immigrant-origin girls and their life prospects cannot be under-stated. Today, more than 40 % of women and girls who live in Canada were themselves or have a parent who was born abroad. Structural inequities are still evident in unemployment figures nationally, with unemployment among women born in Canada sitting at 5.0 %, while among women born abroad it is 9.3 % [66], and 13.2 % in Quebec [67]. This is notable given that women born outside of Canada are more educated than Canadian-born women, though the former are significantly more likely to live in poverty (23 % versus 16 %) [68]. Signalling the role that migration itself may play in this scenario, this source indicates that the figure reaches 35 % among those having arrived to Canada in the past five years. As if this were not significant enough to indicate structural inequities impacting quality of life among immigrant women in Canada, 42 % of female immigrant children under 15 years of age live in poverty, a figure twice the 17 % documented among their non-immigrant counterparts [68].

In this light, findings presented here indicate that the relative school success of South Asian girls is not necessarily accompanied by their greater wellbeing or translated into a greater tendency towards a selective form of acculturation by these girls. Interviews widely suggest that in the context of immigration and poverty, and exposure to a liberal society with arguably greater value put on individualism, the specific race-gender experiences that these girls endure can result in confusion between two cultures, conflicts with parents, high-risk behaviour, and even self-harm. Adding these challenges to non-democratic parental styles suggests that the process of acculturation among South Asian girls, both in terms of maintaining their own ethno-cultural identities and in terms of flexibility to develop a bicultural identity that may lead to optimal outcomes, is challenging. Nevertheless, our data show that some girls appear to have more flexibility in constructing a resilient identity, attempting to bridge the two cultures. That being said, acculturation is a long and complex process requiring separate examination for a fuller, longitudinal picture of the role played by gender. More in-depth and long-term studies are needed to understand how gender shapes the acculturation process of South Asian youth in Quebec. In this respect, the study of the intersection of gender, ethnicity, and class and how this intersection impacts immigrant children's education and adjustment is particularly rich.

## Conclusion

This study reveals the complex interplay of gender with race and socio-economic status to produce relations of domination and subordination – simultaneously among members of an immigrant community and between this community and a host society. Importantly, gendered experiences of South Asian youth in Quebec are not independent of or restricted to conservative cultural values, but are contextual, such that gender roles are crucially shaped by intersections with racial and class identities. Some gender roles described here that may appear disempowering in certain social spheres emerge as advantageous in other contexts. This is evident in the experiences of girls whose socio-educational experiences are constrained by ethno-cultural values, but who nevertheless find means of outperforming male counterparts in school. Intersections of gender with race and class also reveal the concurrent advantages and disadvantages of different gender roles, such that immigrant male youth socialized to be family leaders may be discouraged from excelling in school. This underscores that for South Asian girls in migrant contexts, collectivist and patriarchal values are contextual and not a totalizing form of oppression often presumed by Westerners to limit the life possibilities of such girls. In fact, we see here that traditional gender roles may afford South Asian girls in immigrant contexts the possibility to control certain aspects of their future lives through, for instance, increased potential for school and professional success.

This study is not in a position to determine the extent to which the academic success of these girls will translate into increased access to post-secondary education or increased wellbeing across their lifespan. Nevertheless, the study of the socio-cultural transitions and adaptations to adulthood among South Asian girls could hold insight into the extent to which school success may lead to greater access to the job market, diminished poverty, greater control over reproductive health, and long-term psycho-social wellbeing, even among subsequent generations.

At the very least, avenues of action focused on cultural rapprochement and inter-cultural exchange between the school and the home seem important for promoting the adoption of selective acculturation among immigrant families, and supporting the wellbeing and academic resiliency of their children. This is especially relevant for schools in under-privileged areas, where addressing sexual stereotypes does not happen in isolation of addressing poverty, social segregation and insularity from the host society. One strategy for enhancing the responsiveness and sensitivity of school staff to different familial pressures endured by immigrant children could involve teaching professionals about cultural values among different ethnic groups in their institutions, circumstances in countries of origin, and specific challenges surrounding the migration process for families. Such instruction could, for example, enhance how those from the dominant society understand family honour to play out among South Asian communities, and could enable professionals to recognize forms of flexibility and adaptability that do exist among these groups.

Counselling services focused on prevention of problems related to identity formation could also be helpful to girls of South Asian origin in schools. Our wider data [53] suggest that because of collectivistic values common among South Asian communities, such supports would be more effective if they were offered by female South Asian origin professionals. We observe potential for this to be facilitated through collaborations between schools and South Asian community centers, or by the recruitment of South Asian origin staff within schools. Researchers in the domain of multicultural education recommend that teachers’ ethnicities be carefully considered during the recruitment process, so that educators from similar ethno-cultural groups as students may be more routinely employed [69]. The experience of the first author in interactions with female interviewees further confirms this. As an immigrant woman from Iran, she believes that her experiences promoted deepened intimacy and sharing of stories during interviews, particularly with female students.

Formalizing partnerships between educational institutions and immigrant communities offers promising potential for improved social and educational experiences among immigrant-origin students. For instance, the engagement of parents and families within the school sphere poses to promote shared values and pathways towards reduced risk-taking behaviour among vulnerable students. This is an ongoing challenge in Quebec, where in recent years the Ministry of Education and Higher Learning (Ministère de l’education et enseignment supérieur) and several school boards have sought to partner with community organizations to develop tools to raise awareness about the school system among immigrant families [17]. The lesson here is that meaningfully reaching parents and caregivers involves much more than merely translating resources to meet the linguistic needs of distinct communities. Resources must also appeal to limited time commitments on the part of families located on the low end of the socio-economic spectrum, as well as the possibility of limited literacy skills within a language of origin. The development of more dynamic and less text-based resources also shifts attention beyond innovative strategies for reaching out to families (e.g., films, games, lessons in adult language classes), towards the crucial task of building reciprocal partnerships on the terms that appeal to community members. Similarly, community and media organizations within ethno-cultural networks are not only valuable sites for translating knowledge into more accessible formats, but also key points of departure for a shared project of enhanced community wellbeing.

## Additional file

Additional file 1: Questionnaire (for parents). The Educational Success of Quebec Youth Originating from South Asia: The Impact of Family, Community, and Systemic Factors. (DOCX 172 kb)

## Footnotes

1 Regional sub-groups used by policy-researchers in Quebec for tracking the educational achievement among immigrant-origin students are: East Asia, Southeast Asia, South Asia, North Africa and the Middle East, the Caribbean and sub-Saharan Africa, Eastern Europe, and Central and South America. The determination of these sub-groups was made according to recent immigration profiles and Ministry of Education priorities in educational milieus [16].

2 “Immigrant-origin students” in this paper refers to these two groups.

3 A visible minority is defined by the Canadian government as "persons who are non-Caucasian in race or non-white in colour and who do not report being Aboriginal" [70].

4 To accommodate the influx of students who speak neither French nor English as a first language (in Quebec known as “Allophones”) into French schools, a system of reception classes called *classes d’accueil* - literally welcome classes – has been established. These aim to promote linguistic, academic, and social integration into Quebec society. Students commonly stay in such classes for as many as ten months following their arrival before transitioning into the regular French-language education stream [70, 71].

5 An “under-educated student” is a newly arrived immigrant students who has three years of delay or more in his/her schooling progress ([72], p.10). In 2008-2009, 17 % of Quebec secondary students fell within this category of a significant delay also requiring services from a welcome class.
