# Supplementary material for: Gender at the intersection with race and class in the schooling and wellbeing of immigrant-origin students
Source: BMC Womens Health. 2016 Jul 28;16:47. doi: 10.1186/s12905-016-0328-0 (PMC4964263; doi:10.1186/s12905-016-0328-0)
Supplement: Additional file 1: — Questionnaire (for parents). The Educational Success of Quebec Youth Originating from South Asia: The Impact of Family, Community, and Systemic Factors. (DOCX 172 kb) [file 12905_2016_328_MOESM1_ESM.docx]

Questionnaire
ENGLISH VERSION

**Confidential and Anonymous *Questionnaire***


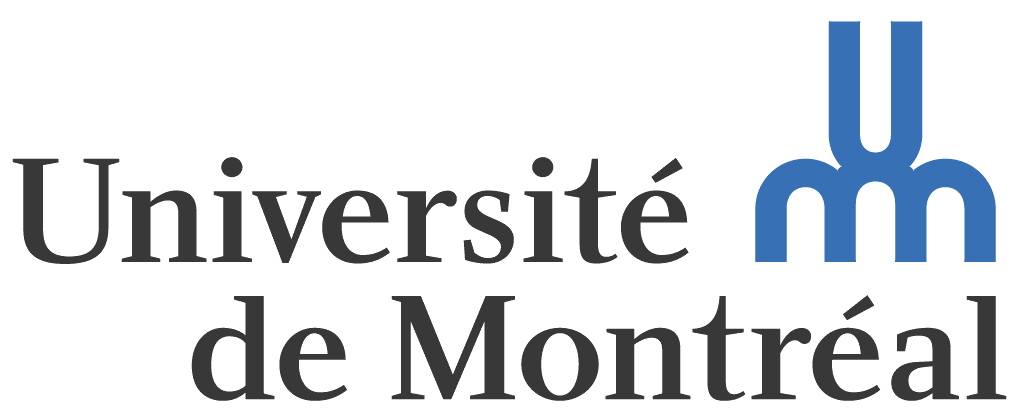


***The Educational Success of Quebec Youth Originating from South Asia: The Impact of Family, Community, and Systemic Factors***

| Dear Parent:  This questionnaire is not a psychological test or exam. It aims to better assess the level of academic performance of secondary students originating from South Asia, and to know your opinions in this regard. Since we also would like to understand what may influence different academic results among students, we will ask general questions your family, your activities, your immigration process, etc.  **You are eligible to participate in this study if you currently have or if you have had, in the past 15 years, a child attending a French public high school in Quebec.**  **If you have more than one child who goes/has gone to a French public high school in Quebec, please fill out this questionnaire with your youngest child in mind.**  **Try to answer all the questions.** There are no right or wrong answers.  Your participation is very important. All the information collected here is confidential and anonymous, and only those people involved in the research project will have access to it. You can therefore share your thoughts freely.  **To keep this anonymity, you must not sign or write your name on this document.**  **INSTRUCTIONS :**  1. Carefully read the questions or statements.  2. Answer the questions by checking a box ❑ or writing a word if the question contains a blank space like this: -----------------.  3.  Pay special attention to what is underlined. These segments provide important instructions or explanations.  4.  You will notice that some questions are at the same time located in the present and past. If your child is still in high school, answer according to your perception of current reality. If (s)he has finished or left high school, answer according to your perception of the time of her/his enrolment in it.  Thank you for your collaboration.  **Researcher and Person in Charge of the Study:**  Mahsa Bakhshaei, Ph.D. student, Department of Educational Administration and Foundations, Université de Montréal.  **Research Supervisor:**  Marie Mc Andrew, Chair holder, Canada Research Chair on Education and Ethnic Relations. Professor, Department of Educational Administration and Foundations, Université de Montréal. |
| --- |

Winter & Spring 2012

**STEP 1: GENERAL INFORMATION**

| 1. Your gender   ❑ F ❑ M | 1. Your English language proficiency   ❑ Very good  ❑ Good  ❑ Average  ❑ Bad  ❑ Very bad |
| --- | --- |
| 1. Your country of origin   ❑ India  ❑ Pakistan  ❑ Bangladesh  ❑ Sri Lanka  ❑ Other *Specify* ……………………………………………. | 1. Your French language proficiency   ❑ Very good  ❑ Good  ❑ Average  ❑ Bad  ❑ Very bad |
| 1. Your mother tongue   ❑ Punjabi  ❑ Tamil  ❑ Bengali  ❑ Urdu  ❑ Sinhalese  ❑ Other *Specify* ……………………………………………. | 1. How many jobs do you currently have?   ❑ I am unemployed  ❑ 1  ❑ 2  ❑ 3  ❑ More |
| 1. How many children do you have?   ❑ 1  ❑ 2  ❑ 3  ❑ 4  ❑ More | 1. What profession did you practice in your home country?   *Specify* *…*……………………………...............  ❑ Not applicable (unemployed) |
| 1. Your level of education   ❑ Primary or secondary education not completed  ❑ Primary school certificate  ❑ Secondary school certificate  ❑ College-level certificate  ❑ University diploma | 1. What is your profession in Quebec?   *Specify* *…*……………………………...............  ❑ Not applicable (unemployed) |

STEP 2: YOUR IMMIGRATION TO CANADA AND QUEBEC

| - 1. In what year did you immigrate to Canada? ........................ | |
| --- | --- |
| - 1. What is your immigration status in Canada?   ❑ Refugee / Refugee claimants  ❑ Citizen  ❑ Permanent resident  ❑ Temporary immigrant  ❑ Other *Specify* .......................................... | |
| - 1. What was your main reason for immigrating?   ❑ Improve my financial status  ❑ Join my family  ❑ Provide my children with better education opportunities  ❑ Difficult political and social circumstances in country of origin (desire to live in a free society)  ❑ Other *Specify* .......................................... | |
| 1. Did you have any choice other than Canada for immigration?   ❑ Yes **🡺 GO TO QUESTION 5**  ❑ No**🡺 GO TO QUESTION 6** | 1. Why did you choose Canada for immigration?   ❑ International reputation of Canada for good living conditions  ❑ Presence of family members in Canada  ❑ Presence of human networks to help with the immigration to Canada  ❑ Potential job opportunity  ❑ Continuing education in Canada  ❑ English language proficiency  ❑ Other *Specify* ......................................... |
| 1. Within Canada, did you choose Quebec in particular over other provinces?   ❑ Yes **🡺 GO TO QUESTION 7**  ❑ No**🡺 GO TO QUESTION 8** | 1. Why did you choose Quebec?   ❑ International reputation of Quebec for good living conditions  ❑ Presence of family members in Quebec  ❑ Immigration accelerated by passing through Quebec  ❑ Potential job opportunity  ❑ Continuing education in Quebec  ❑ French language proficiency  ❑ Other S*pecify* ........................................ |
| 1. Were all your family members accompanying you at the time of immigration to Canada?   ❑ Yes  ❑ No | |
| 1. To what extent do you agree with this statement?   *« Overall, I am pleased to have immigrated to Quebec. »*  ❑ Totally agree  ❑ Somewhat agree  ❑ Neither agree nor disagree  ❑ Somewhat disagree  ❑ Totally disagree | |
| 1. Have you ever thought of immigrating to the United States or to the Anglophone part of Canada?   ❑ Yes  ❑ No | |

STEP 3: YOUR INTEGRATION

| 1. Have you pursued any academic studies or participated in any professional training since arriving to Canada?   ❑ Yes  ❑ No | |
| --- | --- |
| 1. As part of your current job, are you often in contact with South Asian communities?   ❑ Yes  ❑ No  ❑ Not applicable (I don’t work) | |
| 1. To what extent do you agree with this statement?   «*When we arrived in Quebec, the provincial government helped us during the first months of our settlement (e.g. providing information on how to obtain a social insurance number, to rent an apartment, to buy a house, to find a job, etc.).*»  ❑ Yes **🡺 GO TO QUESTION 5**  ❑ No**🡺 GO TO QUESTION 6** | 1. How would you evaluate the services mentioned in the previous question?   ❑ Very good  ❑ Good  ❑ Average  ❑ Bad  ❑ Very bad  ❑ I don’t remember |
| 1. To what extent do you agree with this statement?   *«When we arrived in Quebec, community organizations helped us in the first months of settlement (e.g. providing information on how to obtain a social insurance number, to rent an apartment, to buy a house, to find a job, etc.)*»  ❑ Yes **🡺 GO TO QUESTION 6 & 7**  ❑ No**🡺 GO TO QUESTION 8** | 1. Did these community organizations serve only South Asian immigrants or immigrants in general?   ❑ They only served South Asian immigrants  ❑ They served immigrants in general |
|  | 1. How would you evaluate the services of these organizations?   ❑ Very good  ❑ Good  ❑ Average  ❑ Bad  ❑ Very bad  ❑ I don’t remember |
| 1. To what extent do you agree with this statement?   «*In general, immigrants are well accepted in Quebec*. »  ❑ Totally agree  ❑ Somewhat agree  ❑ Neither agree nor disagree  ❑ Somewhat disagree  ❑ Totally disagree | |
| 1. To what extent do you agree with this statement?   «*In general, immigrants originating from South Asia are well accepted in Quebec*. »  ❑ Totally agree  ❑ Somewhat agree  ❑ Neither agree nor disagree  ❑ Somewhat disagree  ❑ Totally disagree | |

**STEP 4: YOUR CHILD AT SCHOOL**

| 1. How old is your child now? ...................... 2. Her/his gender? ❑ F ❑ M |
| --- |
| 1. In which grade of high school is (s)he now?   ❑ (s)he has finished her/his high school  ❑ Classe d’accueil (welcoming class)  ❑ Secondary 1, 2 or 3  ❑ Secondary 4 or 5 |
| 1. To what extent does/did your child like her/his high school?   ❑ To a great extent  ❑ Somewhat  ❑ Little  ❑ Not at all  ❑ I don’t know |
| 1. In your opinion, what does/did your child like the most about her/his high school?   ❑ Teaching-learning  ❑ Extracurricular and sports activities  ❑ Intercultural atmosphere and activities  ❑ Social relationships with school staff and classmates  ❑ Other  *Specify* ………................................... |
| 1. How was the academic performance of your child during her/his past academic year/last year in high school?   ❑ Very Good  ❑ Good  ❑ Average  ❑ Weak  ❑ Very weak  ❑ I don’t remember |

**STEP 5: YOU AND YOUR CHILD’S SCHOOL**

| 1. Ideally, what is the highest level of education you desire your child to accomplish?   ❑ No degree is required  ❑ High school certificate  ❑ College diploma  ❑ University degree |
| --- |
| 1. In general, what was your main criterion for selecting your child’s high school?   ❑ Reputation of the school and its admission requirements  ❑ Language assistance services  ❑ Proximity of the school to the house  ❑ Presence of students originating from South Asia  ❑ Other *Specify*.............................................. |
| 1. How often do/did you communicate with the school staff of your child’s high school?   ❑ Frequently  ❑ Occasionally  ❑ Rarely  ❑ Very rarely  ❑ Never |
| 1. How is/was the level of the communication of your child’s high school with you?   ❑ Very good  ❑ Good  ❑ Barely acceptable  ❑ Poor  ❑ Very poor |
| 1. To what extent do you agree with this statement?   « *My child goes/went to community organizations that help(ed) her/him with her/his school work »*  ❑ Yes🡺 GO TO THE QUESTIONS 6 & 7  ❑ No 🡺 GO TO THE NEXT PAGE |
| 1. Do these organizations serve all immigrants, or specifically South Asian communities?   ❑ They serve all immigrants  ❑ They specifically serve South Asian communities |
| 1. How would you evaluate the services of these organizations?   ❑ Very good  ❑ Good  ❑ Barely acceptable  ❑ Poor  ❑ Very poor  ❑ I don’t remember |

**STEP 6: YOUR OPINION ON QUEBEC SCHOOLING AND EDUCATION**

| 1. To what extent do you agree with the following statements?    1. « *I am very pleased with my child’s attendance in a French school*. »   ❑ Totally agree  ❑ Somewhat agree  ❑ Neither agree nor disagree  ❑ Somewhat disagree  ❑ Totally disagree   - 1. « *I am very pleased with the grade my child was placed in when (s)he arrived in the Quebec school system?*»   ❑ Totally agree  ❑ Somewhat agree  ❑ Neither agree nor disagree  ❑ Somewhat disagree  ❑ Totally disagree  ❑ Not applicable (My child was born in Canada)   - 1. « *Upon the arrival of my child in high school, (s)he* *and I had generally been well-informed about the school’s regulations, curriculum and programs* »   ❑ Totally agree  ❑ Somewhat agree  ❑ Neither agree nor disagree  ❑ Somewhat disagree  ❑ Totally disagree   - 1. « *The French language courses (classes d’accueil or welcoming classes) are/were well-adapted to the needs of my child*. »   ❑ Totally agree  ❑ Somewhat agree  ❑ Neither agree nor disagree  ❑ Somewhat disagree  ❑ Totally disagree  ❑ Not applicable  ❑ Not applicable (My child was born in Canada)   - 1. « *The duration of the French language courses (classes d’accueil or welcoming classes) was enough for my child*. »   ❑ Totally agree  ❑ Somewhat agree  ❑ Neither agree nor disagree  ❑ Somewhat disagree  ❑ Totally disagree  ❑ Not applicable (My child was born in Canada)   - 1. « *I think that overall my child has enough opportunities to be in contact with French-speaking students*. »   ❑ Totally agree  ❑ Somewhat agree  ❑ Neither agree nor disagree  ❑ Somewhat disagree  ❑ Totally disagree   - 1. « *It’s good that my child in Quebec schools is in contact with those of different ethnicities* »   ❑ Totally agree  ❑ Somewhat agree  ❑ Neither agree nor disagree  ❑ Somewhat disagree  ❑ Totally disagree   - 1. « *Overall, the teachers of my child have/had a fair and just attitude towards my child and have no prejudice against their ethnicity* »   ❑ Totally agree  ❑ Somewhat agree  ❑ Neither agree nor disagree  ❑ Somewhat disagree  ❑ Totally disagree | |
| --- | --- |
| 1. In general, how would you describe teachers’ expectations regarding the academic performance of your child?   ❑ Very high  ❑ Above average  ❑ Average  ❑ Below  ❑ Very low | |
| 1. To your knowledge, is there/have there ever been any tensions or clashes between ethnic or linguistic groups in your child’s school?   ❑ Yes **🡺 GO TO QUESTION 4**  ❑ No**🡺 GO TO THE NEXT PAGE** | 1. Has your child ever been involved in such tensions?   ❑ Yes  ❑ No |

**STEP 7: RECONCILIATION OF YOUR FAMILY VALUES WITH
THE VALUES OF QUEBEC SCHOOL**

| 1. On the scale 1 to 5, to what extent do you agree with the three following statements? 2. « *I am happy that the Quebec education system promotes the development of individualism in our children* »   1 2 3 4 5  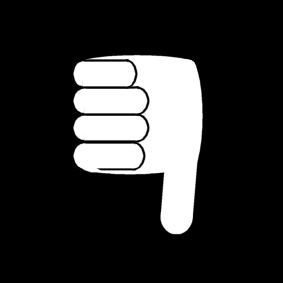 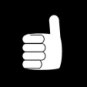   1. « *I totally agree that the Quebec education system encourages our daughters to behave more freely and independently* »   1 2 3 4 5  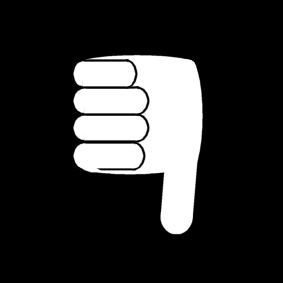 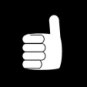   1. « *I am happy that the Quebec education system encourages children to establish equal relationships with their elders. »*   1 2 3 4 5  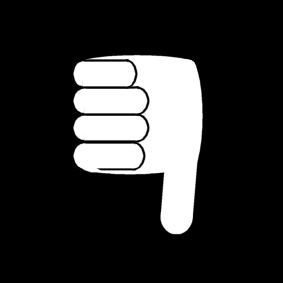 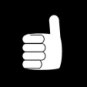 | |
| --- | --- |
| 1. Have you ever seen in one of your child(ren)’s textbooks any representations of your home country or the South Asian community that seemed to you inappropriate?   ❑ Yes  ❑ No  ❑ I don’t know | |
| 1. Have you ever asked the school board to accommodate your child(ren)’s needs for religious or traditional practices?   ❑ Yes 🡺 GO TO QUESTION 4  ❑ No🡺 the questionnaire is finished! | 1. In general, were you satisfied with the response of the school?   ❑ To a great extend  ❑ Some what  ❑ Very little  ❑ Not at all  ❑ I don’t remember |

*Thank you!*
